# Reduced Working Memory Mediates the Link between Early Institutional Rearing and Symptoms of ADHD at 12 Years

**DOI:** 10.3389/fpsyg.2016.01850

**Published:** 2016-11-24

**Authors:** Florin Tibu, Margaret A. Sheridan, Katie A. McLaughlin, Charles A. Nelson, Nathan A. Fox, Charles H. Zeanah

**Affiliations:** ^1^Institute of Child DevelopmentBucharest, Romania; ^2^Department of Psychology and Neuroscience, University of North Carolina, Chapel HillNC, USA; ^3^Department of Psychology, University of Washington, SeattleWA, USA; ^4^Harvard Medical School – Boston Children’s Hospital – Harvard Center on the Developing Child – Harvard Graduate School of Education, BostonMA, USA; ^5^Department of Human Development and Quantitative Methodology, University of Maryland, College ParkMD, USA; ^6^Department of Psychiatry and Behavioral Sciences, Tulane University School of Medicine, New OrleansLA, USA

**Keywords:** children, institutionalization, executive functioning, working memory, ADHD

## Abstract

Children who are raised in institutions show severe delays across multiple domains of development and high levels of psychopathology, including attention deficit/hyperactivity disorder (ADHD). Low performance in executive functions (EFs) are also common in institutionally reared children and often do not remediate following improvements in the caregiving environment. ADHD symptomatology also remains elevated even after children are removed from institutional care and placed in families. We investigate whether poor EF is a mechanism explaining elevated rates of ADHD in children reared in institutional settings in the Bucharest Early Intervention Project (BEIP). In the current study, we examine the potentially mediating role of poor EF in the association between institutionalization and symptoms of ADHD at age 12 years. A total of 107 children were assessed with the Cambridge Neuropsychological Test Automated Battery (CANTAB) on working memory, set-shifting and planning. We also obtained concurrent teacher reports on their levels of ADHD symptoms (inattention and impulsivity separately). Institutionalization strongly predicted elevations in symptoms of inattention and impulsivity at age 12 years (*p*s < 0.01). Indices of working memory and planning were also associated with ADHD after controlling for potential confounders (*p*s < 0.03). Mediation analyses revealed that poor working memory performance mediated the link between exposure to early institutionalization and higher scores of both inattention and impulsivity. These results replicate and extend the findings that we reported in the BEIP sample at age 8 years. Together, they suggest that compromised working memory is a key mechanism that continues to explain the strikingly high levels of ADHD in late childhood among children institutionalized in early life. Interventions targeting working memory may help to prevent ADHD among children exposed to institutional care.

## Introduction

Considerable evidence indicates that early adverse environments can render children vulnerable to various psychiatric disorders that develop later in life (Green et al., 2010; McLaughlin et al., 2012). The link between adverse environments and the onset of psychopathology and other developmental problems is mediated, in part, by disruptions in brain structure and function (McLaughlin et al., 2014a; Sheridan and McLaughlin, 2014; Teicher and Samson, 2016). A typical neural development is particularly likely when exposure to adversity occurs during infancy and early childhood, a period of heightened neural sensitivity to environmental inputs of numerous kinds when the trajectory of brain development is tuned based on the environment the child experiences (Nelson et al., 2009). Early adaptations to an adverse environment can produce lasting changes in cognition and enduring deficits.

A particularly egregious form of early adversity is institutional rearing, which currently impacts approximately eight million children around the world (Save the Children UK, 2009). Institutionalization represents an extreme form of psychosocial and sensory deprivation with a profound impact on multiple aspects of development, including IQ (Nelson et al., 2007), attention (Pollak et al., 2010; Loman et al., 2013), and executive functions (EFs). With regard to EF, there is accumulating evidence to suggest that working memory (Bauer et al., 2009; Bos et al., 2009; Pollak et al., 2010; Hanson et al., 2013; Loman et al., 2013), inhibitory control (Colvert et al., 2008; Bruce et al., 2009; McDermott et al., 2013), error monitoring (Troller-Renfree et al., 2016), and set-shifting (Hanson et al., 2013) are all negatively impacted by early institutionalization. For other EFs, however, such as planning, the findings have been somewhat mixed, with most prior studies reporting negative findings (e.g., Bos et al., 2009; Pollak et al., 2010; Bick et al., in press).

These disruptions in EF might represent a developmental pathway linking early institutionalization to the onset of some forms of psychopathology. The prevalence of mental health problems is unusually high in children reared in institutions, especially attention deficit/hyperactivity disorder (ADHD; Stevens et al., 2008; Zeanah et al., 2009; Humphreys et al., 2015). Current evidence suggests that for young children reared in institutions, adoption or foster care placement does not lead to attenuation in signs of ADHD (Zeanah et al., 2009; Rutter et al., 2010; Humphreys et al., 2015) or EF performance (Colvert et al., 2008; Bos et al., 2009; McDermott et al., 2013; Bick et al., in press), unless the children were removed before 6 months of age (Rutter et al., 2010). Moreover, recent evidence emerging from the English Romanian Adoptees (ERA) study with children adopted in the UK from Romanian orphanages shows persistent ADHD symptomatology through early adulthood (Kennedy et al., 2016), with new cases being diagnosed with ADHD beyond age 20 years. These findings suggest a persistent course of ADHD among children raised in deprived early environments, even after intensive psychosocial intervention, and highlight the need for identifying mechanisms that lead to the very high prevalence of this phenotypic variant of ADHD. Here, we examine the role of disruptions in EF as a mechanism explaining persistent ADHD symptoms among children reared in deprived institutions in early life.

Mechanisms linking institutionalization to ADHD have included multiple manifestations of atypical brain structure and function (McLaughlin et al., 2010, 2014b). However, it is likely that these disruptions in neural development correspond to patterns of atypical cognitive development that might also contribute to the onset of ADHD among children reared in deprived environments. Neuropsychological links between institutional rearing and ADHD have also been demonstrated, including poor inhibitory control (Colvert et al., 2008) and, most recently, we found that working memory and response inhibition mediated the link between institutionalization and two dimensions of ADHD, inattention and impulsivity, among children aged 8 years (Tibu et al., 2016). To the best of our knowledge, no other studies to date have tested the role of EF as a mechanism linking institutional rearing to ADHD symptomatology.

In the current study, we aimed to replicate and extend our previous findings at age 8 by exploring the potentially mediating role of EFs in the association between institutionalization and symptoms of ADHD at age 12. Given ongoing development of EFs during early adolescence (Best and Miller, 2010), and given that ADHD symptoms often decrease in severity during the transition to adolescence (Willoughby, 2003), we were interested in whether EFs would continue to explain the link between early institutionalization and ADHD in this developmental period. Moreover, a recent review on EF findings from studies conducted with previously institutionalized children indicates some contradictory results during late childhood and the need to gather more evidence as children enter adolescence (Merz et al., 2016). Based on BEIP findings at earlier assessments, we expected that children with histories of institutionalization would have increased ADHD symptoms and decreased performance on the EF tasks (Bos et al., 2009; Humphreys et al., 2015; Bick et al., in press). We also expected to find links between executive EF abilities and ADHD symptoms. Furthermore, we investigated whether elevations in ADHD symptoms in the institutionalized children were mediated by differences in EF abilities. To test our hypotheses we included behavioral measurements of multiple EFs (i.e., working memory, set-shifting, and planning) and used teacher reports of ADHD symptomatology.

## Materials and Methods

### Participants

The study participants were children from our longitudinal investigation (Bucharest Early Intervention Project, BEIP) who were recruited in infancy from institutions in Bucharest, Romania, and randomized to either a care-as-usual-group (CAUG) who continued to live in the institutions, or a foster care group (FCG) whom we placed in foster families that we recruited and offered support until children were 42 months old. A third community control group of never institutionalized (NIG) children was recruited at the same time with the institutionalized children through General Practitioner offices. At 12 years, 107 participants in the BEIP were assessed with the Cambridge Neuropsychological Test Automated Battery (CANTAB) on working memory, set-shifting, and planning and we also obtained teacher reports on their levels of ADHD symptoms (with separate measurements for inattention and impulsivity).

Demographic characteristics for the participants in the two groups (i.e., with and without exposure to institutionalization) are presented in **Table 1** and show significant differences in birth weight. Mean birth weight in the institutionalized children was significantly lower than in the community controls (mean difference = -494 grams; *t*(95) = -6.58; *p* < 0.001). Therefore we included birth weight as a covariate in all subsequent analyses.

**Table 1:** Demographic characteristics of the study participants (*N* = 107).

	EIG (*N* = 73)	NIG (*N* = 34)	Group difference
Age at testing in years (*SD*)	12.69 (0.55)	12.78 (0.49)	ns
Birth weight in grams (*SD*)	2758 (595)	3255 (409)	*t* = -4.10; *p* < 0.001
Gender			
Males	40	18	ns
Females	33	16	
Ethnicity			
Romanian	38	33	χ^2^(2) = 20.49; *p* < 0.001
Rroma	26	1	
Other/Unknown	9	0	

*Abbreviations: EIG, ever institutionalized group; NIG, never institutionalized group.*

### Measures

#### ADHD

We obtained teacher reports on children’s symptoms of inattention and hyperactivity using the Health and Behavior Questionnaire (HBQ; Boyce et al., 2002; Essex et al., 2002), a questionnaire with high reliability and validity that has been used extensively with school-aged children (Essex et al., 2002; Lemery-Chalfant et al., 2007). The teacher version of the HBQ was used in the BEIP previously at age 8 years (Tibu et al., 2016), as well as in other studies with formerly institutionalized children (Wiik et al., 2011; Pitula et al., 2014). Symptoms are rated on a 3-point Likert scale: 0 (“never or not true”), 1 (“sometimes true”) or 2 (“often or very true”), with higher scores indicating more severe symptomatology. There are 15 items in the HBQ for assessing ADHD (six items for inattention and nine items for impulsivity).

#### Executive Functioning

The CANTAB was used to measure working memory, planning, and set-shifting. The CANTAB contains behavioral subtests that have been widely used with typically developing children, at-risk children, children with ADHD, and adults (Nigg, 2001; Fried et al., 2015). The CANTAB has been validated extensively on samples of school-age children and has been found to discriminate well between clinical and non-clinical populations (Luciana and Nelson, 2002). Five CANTAB subtests were administered on a desktop computer with a touch-sensitive computer screen to assess working memory, planning, and set-shifting skills.

Delayed Matching to Sample (DMS) is a short term memory task in which the child is presented with a stimulus pattern and then needs to select a matching pattern from a series of four patterns shown below the stimulus. Trials are either simultaneous (both the stimulus and the four choices are shown on the screen at the same time), with a zero-second delay (the stimulus disappears just before the choices are presented), or with a delays of 4000 or 12000 ms. The main outcome variables are the percentage of correct trials and latency of response for each type of trial.

Paired Associates Learning (PAL) subtest assesses spatial working memory and new learning. Six to eight boxes are presented sequentially on the screen, with some or all containing a different pattern. The patterns are then shown again in the middle of the screen, one at a time and in random order, and the child has to touch the box that contained the pattern. The difficulty increases with the number of patterns contained in the boxes. Outcome variables include stages completed at first trial, total stages completed, and memory score (i.e., number of patterns correctly located after the first trial summed across the stages completed).

Spatial Working Memory (SWM) tests the ability to retain spatial information across a delay and to manipulate remembered items in working memory. A number of 3–8 colored boxes are shown on the screen and the subject is invited, by process of elimination, to find a blue token in each of these boxes and use it to fill up an empty column on the right of the screen. Variables of interest are total errors (i.e., total number of errors made by the subject during all trials), between errors (i.e., number of times across trials in which the subject revisits a box in which a token has previously been found), within errors (i.e., number of times within a search in which the subject revisits an empty box), and strategy (i.e., presence/absence of organized patterns of search).

Stockings of Cambridge (SOC) is a planning task derived from the Tower of London test (Shallice, 1982). The child views a set of three hanging stockings on the top of the screen that contain colored circles in a given order and another set of stockings on the lower half of the screen containing the same circles but positioned differently across the stockings. The child is instructed to move the circles in the lower display to copy the upper model using as few moves as possible. The difficulty of the trials increases gradually from 2-move problems to more complex models that require five moves to solve. Key outcomes are number of trials solved in minimum moves, mean number of moves for each level of difficulty, and initial and subsequent thinking times.

Intra-Extra Dimensional Set Shift (IED) assesses rule acquisition and reversal and attention flexibility. Two stimuli (one correct and one incorrect) are displayed on the computer screen. Stimuli initially represent only one category (i.e., shape), then two categories (i.e., line and shape). Participants learn to select the correct categories based on instantaneous feedback from the computer, but the stimuli and/or rules are changed after a specific number of correct responses. The initial shifts in correct stimuli are intra-dimensional (e.g., within the shape dimension) and later become extra-dimensional, requiring a category shift (e.g., from the shape dimension to the line dimension). Performance is assessed based on, among other indicators, number of stages completed and number of errors made.

### Procedure

Ethics approval was obtained from the University of Bucharest’s Ethics Committee, as well as the Institutional Review Boards (IRBs) of the US universities to which the project’s Principal Investigators were affiliated. As the children approached 12 years of age, the families were invited to come for assessments to our laboratory in Bucharest. Consent forms were signed by the legal guardians, and verbal and written assent was also obtained from the children. The CANTAB was administered during one of the lab sessions. The HBQs were distributed to the teachers. Both the families and the teachers were compensated commensurate to the amount of time involved and deemed appropriate in consultation with local staff.

### Data Analysis Plan

To test our hypothesis that EF mediated the link between exposure to institutionalization and ADHD, we first sought to establish links between the predictor and each of the two dependent variables (i.e., ADHD inattention and impulsivity scale scores), as well as associations between our proposed mediators (i.e., indices of EF) and both the predictor and the dependent variables. As a final step, we conducted two sets of mediation analyses (one for inattention and one for impulsivity) and calculated mediation effects (i.e., indirect effects) only for the EF variables that were significantly linked to the predictor and the dependent variables following recommendations from Preacher and Hayes (2008). A bootstrapping procedure was used to test the significance of the indirect effects with 5,000 bootstrap samples and 95% confidence intervals to yield more valid estimates of the indirect effects (Preacher and Hayes, 2008). To support mediation, bootstrapped confidence intervals for the indirect effects cannot contain the value of zero. We included the participants’ birth weight and gender as covariates in all these analyses, and statistical significance was evaluated at the 0.05-level, using two-sided tests. Data were analyzed using the application IBM SPSS Statistics version 23.

## Results

### Foster Care Intervention Effects on ADHD and EF

In line with earlier findings in our BEIP sample at age 8 years, we did not see an intervention effect on children’s ADHD scores or their performance on any of the CANTAB tasks at the age of 12 years. Specifically, children assigned to the FCG did not differ from those in the CAUG on their levels of ADHD symptomatology [*t*(79) = 0.54, *p* = 0.59 for inattention; *t*(79) = 0.50, *p* = 0.62 for impulsivity, which is congruent with parent reports of children’s symptomatology in this sample (Humphreys et al., 2015). Similarly, the two groups did not differ on measurements of working memory, planning, and set-shifting (detailed results not reported here; see Bick et al., in press). Therefore, for the purposes of this paper we combine the FCG and CAUG and examine only two groups, the ever institutionalized group (EIG, *N* = 73) and the never institutionalized group (NIG, *N* = 34). The same approach has been employed in several other BEIP studies that have examined mechanisms linking institutionalization to elevations in ADHD (McLaughlin et al., 2010, 2014b; Slopen et al., 2012; Tibu et al., 2016).

### Exposure to Institutionalization and Child Outcomes

History of institutional rearing predicted scores on both ADHD scales. As shown in **Table 2**, children with history of institutionalization had markedly higher levels of inattention, *F*(1,105) = 19.93, *p* < 0.001, and impulsivity, *F*(1,104) = 8.80, *p* = 0.004, compared to children in the community group.

**Table 2:** Institutionalization status and attention deficit/hyperactivity disorder (ADHD) scores.

	EIG	NIG	Group difference^∗^
Inattention (*SD*)	0.86 (0.55)	0.28 (0.36)	*F* = 19.93; *p* < 0.001
Impulsivity (*SD*)	0.68 (0.52)	0.31 (0.31)	*F* = 8.80; *p* = 0.004

*^∗^Analyses control for birth weight and gender.*

*Abbreviations: EIG, ever institutionalized group; NIG, never institutionalized group.*

Children exposed to institutional rearing had worse performance than those who had never been institutionalized on multiple EF indices in each of the five CANTAB tasks, including all three tests of working memory, planning, and set-shifting (detailed results not reported here; see Bick et al., in press).

Given the disproportionately high number of Rroma children among the EIG compared to the NIG, we tested the potentially confounding role of ethnicity in the link between exposure to institutionalization and ADHD and EF scores. All these associations were unchanged when the Rroma children were removed from the sample.

### Links between ADHD Symptoms and EF Performance

**Table 3** shows the associations of inattention and impulsivity with each of the EF variables that were associated with institutionalization. Performance on the EF tasks was consistently associated with inattention and impulsivity, such that worse performance was associated with higher levels of symptoms. The one exception was the set-shifting task, in which performance was not associated with inattention and impulsivity.

**Table 3:** Correlations between ADHD and EF variables.

	ADHD inattention	ADHD impulsivity	DMS percent accuracy 12000 ms	PAL stages completed on first trial	PAL first trial memory score	SWM strategy	SWM total errors	SOC problems solved in minimum moves	IED stages completed	IED total errors
ADHD inattention	1	0.79^∗∗∗^	-0.31^∗∗∗^	-0.17	-0.26^∗∗^	0.37^∗∗∗^	0.47^∗∗∗^	-0.32^∗∗∗^	-0.11	0.11
ADHD impulsivity		1	-0.29^∗∗^	-0.19^∗^	-0.25^∗∗^	0.35^∗∗∗^	0.41^∗∗∗^	-0.30^∗∗^	-0.15	0.16
DMS percent accuracy 12000 ms			1	0.44^∗∗∗^	0.41^∗∗∗^	-0.26^∗∗^	-0.39^∗∗∗^	0.14	0.21^∗∗^	-0.28^∗∗^
PAL stages completed on first trial				1	0.78^∗∗∗^	-0.25^∗∗^	-0.39^∗∗∗^	0.27^∗∗^	0.17^∗^	-0.26^∗∗^
PAL first trial memory score					1	-0.19^∗^	-0.31^∗∗∗^	0.32^∗∗∗^	0.11	-0.18^∗^
SWM strategy						1	0.66^∗∗∗^	-0.27^∗∗^	-0.09	0.23^∗∗^
SWM total errors							1	-0.44^∗∗∗^	-0.15	0.23^∗∗^
SOC problems solved in minimum moves								1	0.02	-0.12
IED stages completed									1	-0.63^∗∗∗^
IED total errors										1

*^∗^*p* < 0.05, ^∗∗^*p* < 0.01, ^∗∗∗^*p* < 0.001.*

*Abbreviations: ADHD, Attention Deficit/Hyperactivity Disorder; EF, Executive Functioning; DMS, Delayed Matching to Sample; PAL, Paired Associates Learning; SWM, Spatial Working Memory; SOC, Stockings of Cambridge; IED, Intra/Extra Dimensional set shift.*

After controlling for birth weight and gender, several working memory and planning indices continued to predict ADHD levels. Specifically, associations remained significant between inattention and DMS percent accuracy 12000 ms (β = -0.22; *p* = 0.024), PAL first trial memory score (β = -0.23; *p* = 0.016), SWM strategy score (β = 0.30; *p* = 0.002), SWM total errors (β = 0.42; *p* < 0.001), and SOC problems solved in minimum moves (β = -0.29; *p* = 0.003), and also between impulsivity and PAL first trial memory score (β = -0.22; *p* = 0.023), SWM strategy score (β = 0.32; *p* = 0.001), SWM total errors (β = 0.38; *p* < 0.001), and SOC problems solved in minimum moves (β = -0.26; *p* = 0.007).

### Mediation Analyses

**FIGURE 1:**
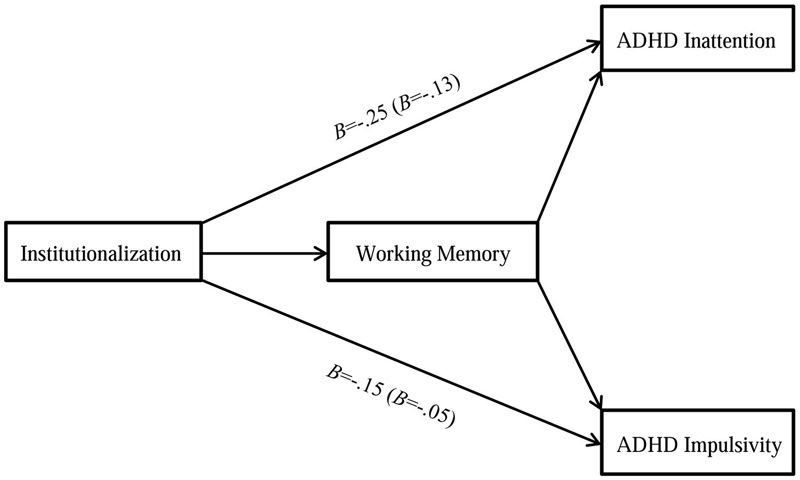
**Full mediation models linking exposure to institutionalization to inattention and impulsivity through working memory**.

In our final sets of analyses we tested our main hypotheses; namely that poor EF performance explained the link between exposure to institutionalization and elevated ADHD symptoms. To do that, we tested two mediation models, one for each of the two dependent variables, in which we included the institutionalization status as the predictor, EF indices as mediators and birth weight and gender as covariates. For each model, we tested separately whether the indirect effect of institutionalization on ADHD symptoms through working memory and planning was statistically significant. These findings are presented jointly in **Figure 1**.

In predicting inattention, we first included as mediators the four working memory variables jointly (i.e., DMS percent correct at the 12,000 ms trials, PAL first trial memory score, SWM strategy score, and SWM total errors), and then the planning index (i.e., SOC problems solved in minimum moves) as sole mediator. The indirect effect of exposure to institutionalization on inattention through the working memory indices was significant (95% CI: -0.24, -0.04). The total effect of institutionalization in predicting inattention was attenuated by 48.6% to non-significance level (*B* = -0.13; *p* = 0.067) when these working memory variables were included as mediators. By contrast, when the planning index was included as a mediator, the indirect effect of institutionalization on inattention was not statistically significant (95% CI: -0.07, 0.00). These findings suggest that the link between exposure to institutionalization and inattention is mediated by working memory, and not explained by planning.

Likewise, in predicting impulsivity, we first included as mediators three working memory variables jointly (i.e., PAL first trial memory score, SWM strategy score, and SWM total errors), then the planning index (i.e., SOC problems solved in minimum moves) as sole mediator. The indirect effect of exposure to institutionalization on impulsivity through the working memory indices was significant (95% CI: -0.23, -0.04). The total effect of exposure to institutionalization in predicting impulsivity was attenuated by 66.5% to non-significance level (*B* = -0.05; *p* = 0.43) with the inclusion of the working memory indices as joint mediators. In contrast, the indirect effect of institutionalization in the prediction of impulsivity when the SOC variable was entered as mediator was statistically non-significant (95% CI: -0.07, 0.00). These results suggest that the link between exposure to institutionalization and impulsivity is, as with inattention, explained by performance in working memory and cannot be attributed to the children’s planning ability.

## Discussion

Children with histories of institutional rearing continue to display elevated symptoms of ADHD and poor EF performance in the pre-pubertal period. In the current study, we show that EF mediates the link between institutionalization and symptoms of both inattention and impulsivity, which provides support for the hypothesis that low cognitive abilities represent a distinct pathway through which early institutional care exerts a persistent impact on mental health problems. As we expected, children reared in institutions had higher ADHD symptomatology and worse performance across EF tasks compared to a control group (for detailed results on the EF, see Bick et al., in press). We found that poor working memory specifically acted as a mediator in the associations of institutional rearing with both inattention and impulsivity, highlighting the key role played by this higher-order cognitive function in explaining how institutionalization can contribute to persistent ADHD. Working memory appears to be one factor explaining the link between institutional rearing and ADHD, although clearly other mechanisms are likely to be involved.

The results in the current study replicate and extend findings from other studies that have tested samples of previously institutionalized and adopted children of comparable ages to the children in our study. Specifically, our finding that previously institutionalized children exhibit markedly elevated rates of inattention and impulsivity as reported by their teachers is consistent with caregiver reports obtained through a structured interview in the BEIP (Humphreys et al., 2015) and with other studies showing high ADHD symptoms among children who were institutionalized in early life and later adopted into families in Western Europe (Stevens et al., 2008; Vorria et al., 2014; Kennedy et al., 2016) and North America (Gunnar et al., 2007; Wiik et al., 2011). Our current results extend findings from this sample at earlier ages, which also observed high levels of ADHD symptoms (Zeanah et al., 2009; McLaughlin et al., 2014b; Tibu et al., 2016), by documenting persistently elevated levels of ADHD throughout childhood and into early adolescence following early institutionalization. It is possible that the trajectories of ADHD symptoms in children who grew up in institutions follow a different trajectory compared to ADHD seen in family-reared populations in that the levels of symptoms remain high, sometimes even becoming higher as children enter young adulthood (Kennedy et al., 2016).

Additionally, we replicate and extend our previous findings on the mediating role of EF in the link between early institutional care and ADHD symptomatology in middle childhood (Tibu et al., 2016). Here, we found again that compromised working memory ability is a key mechanism that explains elevated ADHD symptoms in children who had been exposed to institutionalization. These findings reveal a cognitive mechanism that explains the link between institutionalization and ADHD, emphasizing the pervasive influence of early institutionalization on cognitive development. Interestingly, experiences of institutionalization early in life appear to impact cognitive function even in domains associated with areas of the brain known to exhibit a protracted developmental trajectory into adolescence. Working memory is supported by prefrontal and superior parietal cortex function and shows a protracted developmental trajectory into late adolescence (Thomason et al., 2009; Finn et al., 2010; Peverill et al., 2016). Disruptions in spatial working memory have previously been observed among children with ADHD (Westerberg et al., 2004), as well as atypical neural structure and function in fronto-parietal networks (Giedd et al., 2001; Castellanos et al., 2002).

How exactly reduced working memory exerts influence on the development of ADHD is difficult to answer given that multiple pathways resulting from interplays between individual predispositions and environmental adversities are likely to be involved (Sonuga-Barke and Halperin, 2010) and institution-related ADHD appears to differ substantially from ADHD in typical populations (Kennedy et al., 2016). It is possible that institutional care in the first years of life disrupts profoundly neural structures (e.g., the prefrontal cortex; PFC) that are responsible for the on-going development of memory and attention (Nelson et al., 2011), which in turn, can result in abnormally high levels of inattention and impulsivity. The PFC is an area of the brain that has been shown in numerous studies to be influenced by deprived early environments (McLaughlin et al., 2014a,b; Sheridan and McLaughlin, 2014). Specifically, children raised in deprived environments exhibit reductions in cortical thickness in the PFC as well as the parietal cortex (McLaughlin et al., 2014b; Mackey et al., 2015). This pattern is consistent with conceptual models of deprivation, which argue that accelerated synaptic pruning occurs in the PFC and other areas of association cortex when the environment is lacking in cognitive and social stimulation (McLaughlin et al., 2014a; Sheridan and McLaughlin, 2014). This accelerated cortical thinning may contribute to the development of poor working memory and, ultimately, ADHD. Indeed, the association between institutional rearing and ADHD has been shown to be explained, in part, by exaggerated cortical thinning and a pattern of blunted cortical activity, including in the PFC and parietal cortex (McLaughlin et al., 2010, 2014b). It is likely that early deprivation associated with institutionalization alters the development of fronto-parietal networks that underlie working memory, and that this altered neural circuitry ultimately contributes to behavioral problems in the form of elevated ADHD symptoms. Clearly, more research is needed into identifying specific cortical and subcortical abnormalities impacting EF and ADHD in the institutionalized children.

Planning ability, although impacted by institutionalization and linked to ADHD, was not a construct that explained ADHD symptoms in our current study, which is consistent with our BEIP findings at age 8 years. It is possible that planning, which is thought to be a more sophisticated problem-solving cognitive skill, is not affected by institutionalization in the same manner as other more fundamental EFs like working memory. There is evidence suggesting that working memory and inhibition are more central processes that help toward the development of most of the other EFs (Senn et al., 2004), and it may be that disruptions that occur during early childhood impact on the more basic functions, which may in turn lead to signs of ADHD in the first years of life.

There are several noteworthy clinical and theoretical implications regarding the findings in the current study. First, caregivers and specialists should be informed that many of the children who had been reared in institutions will exhibit high levels of ADHD symptoms that are likely to persist throughout childhood and beyond. Foster and adoptive parents need to become aware of the enduring neurocognitive deficits and impulsive/inattentive manifestations that many of these children will show across settings. Second, there is potential for interventions to be designed that may decrease these symptoms by improving children’s cognitive abilities, at least their working memory. Indeed, there are programs that have proved their efficacy in addressing ADHD problems through working memory training (Klingberg et al., 2005; Gray et al., 2012; van der Donk et al., 2015), although there is no evidence yet on how effective such interventions might be with institutionalized children. Lastly, developmental models of ADHD linked to institutionalization should not omit testing working memory and possibly other EFs too, among its contributing factors.

The strengths of the current study include a longitudinal sample from an RCT with a rarely studied population that had been exposed to institutional rearing, some from as early as 6 months of age, a robust mediation model that allows testing a causal understanding on ADHD while controlling for confounders, and good retention rates in a sample of highly vulnerable adolescents recruited more than a decade ago.

Our study should also be discussed in terms of its limitations. First, we included concurrent measurements of EF and ADHD, which may pose some limitations in inferring causality. Second, we made use of questionnaires as opposed to interviews for measuring ADHD symptoms. Nevertheless, teacher reports are reliable instruments in measuring levels of symptomatology, particularly of externalizing nature (Cai and Kaiser, 2004) and have been used before in studies with institutionalized children and adolescents. Finally, we were not able to account for the possible influence of other factors (e.g., prenatal, genetic risks, or medical illnesses during the gestational or early infancy periods, etc.) that might have played a direct or interactive role in the link between exposure to institutionalization and ADHD and which should be examined in future studies. Interestingly though, evidence from the ERA study indicates that children removed from institutions before age 6 months had comparable ADHD scores with control participants (Rutter et al., 2010), suggesting that the high rates of ADHD in post-institutionalized children do not simply reflect prenatal and genetic factors but also the impact of being raised in a deprived environment. Certainly, prenatal and genetic factors play an important role, and we cannot adjust for their influence in the current study.

This study identified poor working memory as a mediator of the association between institutional rearing and ADHD symptoms of inattention and impulsivity at 12 years of age in children who, during their early childhood, grew up in institutions. Mental health problems, ADHD in particular, are a public health concern at global level and our current findings have the potential to inform specialists on some of the neuropsychological mechanisms to psychopathology during early adolescence so that interventions can be effectively implemented to reduce maladaptation and increase chances of academic success and optimal social and occupational functioning.

## Author Contributions

CN, NF, and CZ designed research; FT performed research; FT, MS, and KM analyzed data; FT, MS, KM, CN, NF, and CZ wrote the paper.

## Conflict of Interest Statement

The authors declare that the research was conducted in the absence of any commercial or financial relationships that could be construed as a potential conflict of interest. The reviewer VS and the handling Editor declared their shared affiliation, and the handling Editor states that the process nevertheless met the standards of a fair and objective review.
